# Intelligent Animation Creation Method Based on Spatial Separation Perception Algorithm

**DOI:** 10.1155/2022/4999478

**Published:** 2022-09-19

**Authors:** Qingbo Meng

**Affiliations:** Qingdao Agricultural University, College of Animation and Media, Qingdao 266109, Shandong, China

## Abstract

In the computer group animation creation technology, the artificial life method of computer animation overcomes the defects of traditional animation creation technology and greatly improves the animation creation efficiency. However, due to the increasing complexity of the animation character modeling technology used in this method, the coupling degree between the models of the animation system is also increasing, which makes the animation creation increasingly difficult. Especially, when the number of characters' increases, the computation will increase rapidly in a nonlinear way, which greatly affects the real-time animation creation and limits the wide application of this method. In this paper, we have conducted an in-depth study and implementation of the design of the animation character model and its implementation technology, analyzed and designed the group animation character model, and designed the space separation perception algorithm to effectively reduce the design difficulty of the character biomechanical model, reduce the amount of computation, and further ensure the real time of large-scale group animation creation. Therefore, the research reduces the coupling between animation system models without reducing the animation effect and real-time performance. It reduces the amount of computer operation, meets the real-time requirements of large-scale group animation creation, and has important significance and value.

## 1. Introduction

As a crystallization of computer graphics and art, computer animation refers to the use of graphics and image processing techniques to generate a series of scenic images with the help of programming or animation creation software [1]. Among them, the current frame is a partial modification of the previous frame, and it is a high technology that has developed at a high speed along with computer hardware and graphics algorithms. It integrates the knowledge of computer science, art, mathematics, physics, and other related disciplines to generate brilliant and continuous virtual real images on the computer, providing a new world for people to fully display their personal imagination and artistic talent. Now it has been widely used in various fields of national economy and social life, which has produced huge economic and social benefits [2]. In the creation of computer animation, group animation creation has been a technical problem that has trapped computer creators.

Traditional animation mainly relies on manual drawing and key frame technology. The limitations of these technologies make the production of group animation consume a lot of manpower, financial resources, and time. It cannot guarantee the intelligence of the role and the authenticity of the group behavior. Therefore, the research of group animation based on intelligent algorithms has gradually become the focus of the majority of scholars. The limitations of traditional animation include too fine division of labor, high equipment requirements, and high cost. The application of traditional animation creation methods has produced many excellent animations, including some animal group animations, but it has some unavoidable limitations such as: (1) it requires a lot of animator's labor. In traditional animation techniques, such as key framing, each character's movement and movement details are designed step by step by highly skilled animators. With the lengthening of the animation, the increase of the number of characters, and the increase of the complexity, the labor of the animators also increases significantly. (2) Lack of autonomy of animated characters: characters driven by key frames or motion data methods lack autonomy, thus reducing the modifiability and interactivity of the animation. Since the real behavior of the animal is influenced by the state of its environment, slight modifications to the animation script require the entire animation to be recreated, and the animated character cannot automatically coordinate with its surroundings. (3) Tedious action detailing: the traditional key frame technique is used to create animated characters with little or no autonomy. Therefore, every movement of the animated character has to be planned one by one by the animator who has complete control over every detail of the animated character. A lot of the animator's energy will be spent on this low level of control [3, 4]. (4) Natural authenticity is difficult to guarantee. It is clear from the above that the realism of animation can only depend on the animator's skill. Since the usual geometric model does not have mechanical properties, it is entirely up to the animator to carve the character. Therefore, the physical correctness of the depicted motion is not guaranteed, and the animated character does not respond to external forces. Unless the animator has a high level of skill, poor visual effects will occur [5, 6].

In addition, if the traditional computer animation creation method creates a virtual natural environment to create a variety of animal groups, animation is very complex and extremely cumbersome, not only in the software design and programming detailed provisions of each animal every sampling moment, every action, every body shape, every posture, but also a specific description of many animals in relation to each other, mutual position, and interaction between the “combination explosion.” The computational complexity will grow at an exponential rate, resulting in a “combinatorial explosion,” and even with a high-speed, high-capacity computer, the animation may not be able to achieve satisfactory results that are realistic [7].

Unlike the traditional “key frame” technology used in computer animation creation, the artificial life method of computer animation is an automatic animation generation method based on the natural life model, which can not only significantly increase the realism and vividness of computer animation and improve the sense of immediacy of animation, but also can effectively improve the creation of animation. It not only can significantly increase the fidelity and vividness of computer animation and improve the sense of immediacy of animation, but also can effectively improve the creation efficiency of animation, significantly reduce the intervention and interference of animation creators to the process of computer animation generation, and reduce the labor intensity of animators so that animators change from the role of “puppet show performer” to the role of “cinematographer” in the virtual nature [8]. The research and implementation of the artificial life method of computer animation integrate the knowledge of computer graphics, artificial intelligence, artificial life, physics, biology, and other disciplines, break through the traditional framework of computer animation, open up a new way of computer animation creation, and provide a powerful method and way for computer animation technology to be truly applied in the market. Its research results also provide a complete research test platform for the research of multi-intelligent body system, complex adaptive system, artificial life, robotics theory, computer vision, machine learning, and other disciplines [9].

Unlike the traditional distributed AI, the intelligent character in the computer-animated artificial life approach not only has various characteristics of the distributed AI discipline, but also has biological appearance and morphological and motor performance capabilities, enhancing the intelligent body as a “living organism” with a certain appearance and morphology that can survive and perform on its own. This shows that the artificial life approach of computer animation is essentially a fusion of the “bottom-up” “emergent” research idea of the “artificial life” discipline [10]. The “intelligent body” research method of artificial intelligence discipline is introduced into the computer animation creation, and each animation character is treated as an enhanced intelligent body according to the real or imagined biological characteristics to give each animation character a certain simple intelligence through the interaction between animation characters to realize the global emergence of group behavior. The artificial life method of computer animation has been proved to be a good and effective method for the creation of computer group animation.

As animation creation research increasingly focuses on the intelligence of animated characters and the delicacy of their movements, animation character modeling techniques are becoming more and more complex, and the coupling between the models of the animation system is also increasing, which makes animation creation increasingly difficult. For example, the body and action of the animated character have been developed from key frame technology to be generated and driven by biomechanical model, but it is not only theoretically difficult to build a biomechanical model of the animated character, but also the computer computation is very large during the operation [11]. When the number of intelligent characters' increases, its computation will increase nonlinearly and rapidly, which greatly affects the real-time animation creation without a better balance between simplicity and efficiency.

The innovative contribution of this paper is to design a two-layer decomposition group animation character model based on autonomous intelligence under the premise of ensuring the animation effect and real-time performance. By reducing the computing power of the computer, the coupling between animation models is reduced, and the animation production efficiency is improved. And the specific implementation technology of the model is deeply studied and implemented. The design and implementation technology of animation character model are deeply studied and realized, and the group animation character model is analyzed and designed. The spatial separation perception algorithm is designed to effectively reduce the difficulty of designing the character biomechanical model. It reduces the amount of calculation and further ensures the real-time nature of large-scale group animation creation. In the computer group animation creation technology, the artificial life method of computer animation overcomes the defects of traditional animation creation technology and greatly improves the animation creation efficiency.

This paper is organized as follows: Chapter 1 describes the research background of autonomous intelligent body animation creation and the main structure of this paper; Chapter 2 introduces the status of domestic and international research in related fields and summarizes the significance of this paper; Chapter 3 studies and designs a two-level decomposed group animation character model based on autonomous intelligent body and briefly explains the components of the model and the specific functions and implementation techniques of each part.  Chapter 4 designs a spatial separation perception algorithm for the performance deficiencies of the point measurement method in complex scenes using the finite perception characteristics of intelligent characters and proposes the group search optimizer algorithm with Metropolis and predictive (MPGSO) to provide a new method for path planning in complex animation environments.  Chapter 5 tests and analyzes the scheme proposed in this paper.  Chapter 6 summarizes the research content of this paper and provides an outlook on future research directions.

## 2. Related Works

Before the artificial life method of computer animation was proposed, some previous scholars had done some work similar to the creation of artificial life method of computer animation and realized a series of excellent works [12]. Each individual role is an independent participant in its own ecosystem and determines what behavior to take next by sensing local environmental information at a given time to simulate cluster behavior through role interaction. Pla-Castells et al. demonstrated the situation of groups of robots through physics. In Thalmalin's work, virtual characters with simple visual, tactile, and auditory receptors can perform tasks, such as following leaders, greeting each other, and even playing ball games [13].

The intelligence of group animation characters and the subtlety of their movements is a distinctive feature of foreign research in this and related fields [14]. In particular, Cole et al. have been at the forefront of research on computer vision, machine learning, and cognitive modeling based on the “artificial fish” animation framework created by computers [15]. Among them, computer vision research mainly uses computer graphics knowledge to build “integrated visual models” of animated characters to improve the perceptual performance of animated characters when the number of characters in group animation increases and the environment of animated characters is complex and changing; machine learning and cognitive modeling research is mainly used to improve the intelligence of animated characters. Machine learning and cognitive modeling are mainly used to improve the intelligence of animated characters, especially of higher organisms, for the simulation and emulation of advanced animated characters [16].

In addition, the study and application of swarming behavior has become a hot topic in computer animation creation and its related fields. The research on swarming behavior is mainly to study the influence of the speed of information transfer between characters on the vividness of portraying swarming behavior and to use swarming behavior to portray the movement of complex systems similar to the flight of a flock of birds or the swimming of a school of fish. In the virtual reality and entertainment industry, a series of excellent film and television works have been created by combining multi-intelligence research and animation creation [17].

In China, the research on artificial life methods of computer animation and its related fields started late but progressed rapidly [18]. Nowadays, the research of artificial life methods for computer animation combined with multi-intelligent body systems and complex adaptive systems has become the characteristic and hot spot of current computer animation research and creation [19]. Representative works include the self-reproduction model of artificial fish, the study of self-learning methods, the evolutionary model of artificial fish, and the cognitive model implemented by Zoss et al. [20].

This paper combines domestic and international literature to focus on the construction and implementation of the role model from both the structural design of the role model and its specific implementation techniques. The simulation method of group intelligent behavior is studied based on a comprehensive understanding of the research content, ideas, and especially the research methods of the artificial life discipline. Combined with the current animation creation ideas, a two-layer decomposed group animation character model based on autonomous intelligences is creatively designed, and the specific implementation technology of each model is further studied in depth. In the specific implementation technology of the character model, a “spatial separation” algorithm is designed for the perceptual model to improve the perceptibility of the character, which can effectively solve the performance bottleneck of group character animation. In addition, this paper proposes a method of multi-threaded path planning using group search algorithm to address the problems of long search time and poor search capability of traditional path planning algorithms in complex animation environments.

## 3. Group Intelligent Character Animation Model Design

Population animation is a simulation of the behavior of biological groups and is an important part of the study of complex systems. Unlike traditional simulation methods, population behavior simulation now mainly uses the bottom-up “emergent” approach, and current research work is mainly focused on modeling individual group characters. The new generation of computer animation “artificial fish” developed by Dr. Xiaoyuan Tu is known as “Xiaoyuan's fish” due to its great academic value and is cited in the common mathematics textbooks in English-speaking countries.

**Figure 1:**
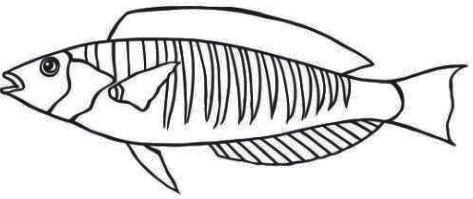
Character model of artificial fish.

As shown in Figure 1, the artificial fish has a fish brain inside the body, and the fish brain itself consists of three control centers: perception center, behavior center, and movement center. The motion system of the artificial fish includes a kinetic model of the fish, effectors, and a set of motion controllers. These motion controllers constitute the motion control objects in the artificial fish brain.

**Figure 2:**
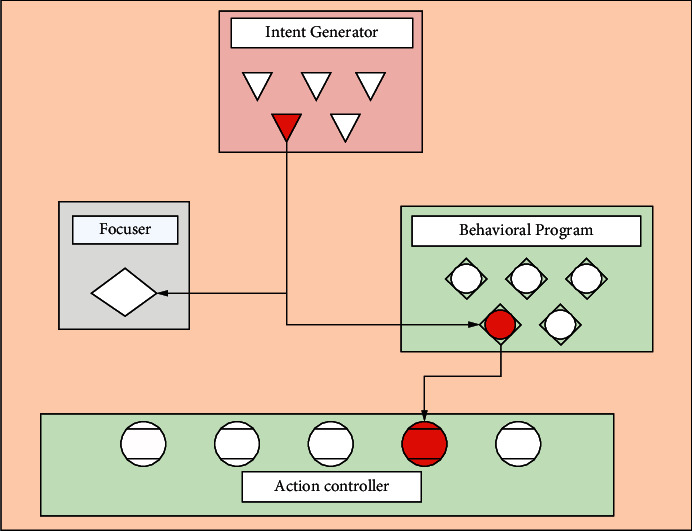
Overall control scheme for artificial fish action selection.

The action selection control scheme for artificial fish consists of two important relationships. One is the relationship between motor controllers and muscles/fins, and the other is the relationship between certain high-level control mechanisms and motor controllers. The action selection process in the behavioral control and action selection scheme of artificial fish consists of two levels: the intention level and the action level. The separation of the intention level and action level makes the design more intuitive and efficient. In particular, since the function of the intention generator is independent of the function of the behavior program, new behaviors can be added to the behavior program list at each step chase. First, the behavior program for the new behavior can be written and tested independently of the intent generator. Second, the intent generator can be modified to fit the new intent and it can also be tested independently. The intent is generated by the intent generator, and the action is manipulated by the focuser and the behavior program. This is shown in Figure 2.

In order to address the defects of the “artificial fish”-like animated character model, such as the difficulty of establishing and controlling, the complexity of constraint relations, the large amount of computation, and the limitation of real-time animation, this paper designs a new group animated character model—a two-layered model based on autonomous intelligences

The two-level decomposition group animation character model based on autonomous intelligences is divided into three models: perception model, behavior decision model, and motion model, which correspond to the perception system, behavior control system, and motion system of the realized animated individual character, respectively. Among them, the behavioral decision model contains two sub-models: motivation model and behavioral decision model. The somatic motion model also contains two sub-models: the overall motion model and the animation model. Compared with the previous model, the model division also includes three parts: perception model, behavioral decision model, and motion model; from this perspective, when creating a group animation work similar to “artificial fishes,” we can use this group animation character model to import a model. In this way, when creating a group animation work like “artificial fish,” we can use this group animation character model and, at any moment, import a mechanism similar to the virtual fish action selection mechanism in “artificial fishes” to realize the flexible swimming of virtual fish. Combined with the created underwater scene and then importing the fish swarming algorithm, you can generate real-time virtual fish movement animation.

Using this intelligent character animation model to create animation, firstly, use the mask skeleton animation technology to create the body of the intelligent character, then take the intelligent character as an independent intelligence, get the virtual environment information through the perception model, change its inner mental state, generate or combine the intention specified by the animator independently, get the current intention through the behavior generation mechanism, choose the appropriate behavior, and finally act with the environment and synthesize. Finally, it works with the environment to generate the “main power,” which acts on the body of the intelligent character to generate the behavioral movement of the intelligent character and combines the behavioral movement of the intelligent character to synthesize the motion animation of the intelligent character, so as to realize the character's intention. Intelligent human animation technology is based on the existing 3D human motion capture data. The purpose of human animation production technology for animation sequence generation is to take the existing human motion data as input. The essence of the above methods is to insert the selected motion sequence into the scene and edit it according to the scene.

## 4. Perceptual Model Research and Implementation

### 4.1. Spatial Separation Perception Algorithm

In animation creation, information about the position and shape of each character in the scene can be obtained by querying the virtual environment. By calculating the distance from the geometric center of each scene and/or character to the center of the eyes of the perceiving subject character, it can be approximated whether they are within the perceptual range or not. For living beings, an object is visible when it is within the perceptual range and is not obscured by other objects. The point measurement method works according to this idea.

There are various implementations of occlusion detection algorithms for different modeling approaches. One of the occlusion detection algorithms is presented below with an artificial fish as an example.

To determine the visibility, the visual receptors were set in the ontological coordinate system (*X*_*f*_, *Y*_*f*_, *Z*_*f*_) on the right side of the artificial fish, with the *x*-axis oriented along the fish's vertebrae and the *y*-axis pointing to the left side of the fish.

To determine whether a point P is visible, a line of sight is drawn from the origin *O* (near *X*_f_ and the fish's mouth) to *P*_0_. To check whether a point *P* is occluded, it is necessary to test whether the line of sight is cut off by other objects (other fish or stationary obstacles) in the field of view of the artificial fish. In complex virtual environments, for obstacles with complex geometry, columns can be used as their “bounding boxes,” and the “bounding box” technique is used to speed up the occlusion detection process.

Let the axis of each cylinder be parallel to the *Z*-axis of the world coordinate system, so the algorithm of measuring the intersection of the line of sight and the cylinder is simple. The coordinates of point 0 and point *P* in the world coordinate system are expressed as (*X*_0_, *Y*_0_, *Z*_0_) and (*X*_*p*_, *Y*_*p*_, *Z*_*p*_), respectively, and the line *OP* can be expressed by the following parametric equation:

(1) $$\begin{array}{c} X=X_{0}+t*\Delta X\text{,}\\ Y=Y_{0}+t*\Delta Y\text{,}\\ Z=Z_{0}+t*\Delta Z\text{.} \end{array}$$

Among them,

(2) $$\begin{array}{c} \Delta X=X_{p}-X_{0}\text{,}\\ \Delta Y=Y_{p}-Y_{0}\text{,}\\ \Delta Z=Z_{p}-Z_{0}\text{.} \end{array}$$

Let *r* denote the radius and *h* denote the height of the cylinder *C*, then its projection on the *XY* plane is a circle *A*, which can be expressed by the following parametric equation:

(3) $$\begin{array}{c} {\left(x-a\right)}^{2}+\left(y-b^{2}\right)=r^{2}\text{,} \end{array}$$

where (*a*, *b*, 0) is the center of circle *A*.

In order to realistically mimic the perceptual capabilities of the swarm intelligent actor, visibility detection of entities in the surrounding environment is performed in each simulation cycle. As mentioned above, the point measurement method consists of two steps: first, field-of-view detection, which checks whether an object is in the field of view of the intelligent actor; if it is, then occlusion detection is performed to check whether it is occluded. In this process, the amount of computation does not increase linearly with the number of objects in the field of view because only a small portion of the objects in the smart character's field of view are detected for occlusion. However, this is not the case for the computation when performing field-of-view detection. If a common algorithm is used, the computational complexity is *O*(*n*) (for each character), where *n* is the number of entities in the character's living environment. The total computation required to perform the field-of-view detection of intelligent characters grows quadratically with the number of entities in the animation. Therefore, when the number of characters in a 3D virtual environment is large, such as in a large-scale combat creation simulation, the field-of-view detection computation will be in a large order of magnitude and a more efficient method for field-of-view detection is desired. Thus, it is clear that how to improve the performance of field-of-view detection is of great value to ensure the real-time performance of large-scale group animation creation.

Notice that the actor can only perceive entities within its perceptual radius *Vr* at each simulation cycle, the perceptual subject. Objects outside the perceptual radius *Vr* are of no value in terms of the final result of perception, other than increasing the computational effort; i.e., the actor has a finite perceptual property. In other words, the actor can only perceive entities in its specific space at a given moment, which is “space-time” coherent. To this end, a new perception algorithm is designed from the perspective of the limited perceptual characteristics of the actors, using the “space-time” coherence to spatially segregate the living environment of the actors, so that each actor perceives only the entities in a specific space in each simulation cycle, i.e., only the “potentially visible.” The implementation is achieved by combining each perceptual subject with the other perceptual subjects. This is achieved by having a table of “potentially visible objects” for each perceptual subject, which is added to the “potentially visible objects” table of the perceptual subject when the character enters a specific space. This table is generated at the beginning of the animation and is updated intermittently. At each animation time step, assuming that the maximum velocity of the character's movement is limited, only the entities in the “potentially visible objects” table need to be detected if they enter the field of view of the perceptual subject, and no other entities need to be detected.

### 4.2. Group Search Optimizer Algorithm with Metropolis and Predictive

A method of multi-threaded path planning using group search algorithm is proposed to address the problems of long search time and poor search capability of traditional path planning algorithms in complex animation environments. The method firstly introduces the simulated annealing algorithm into the search mode to overcome the problem that the algorithm is easy to fall into local optimum; under each control parameter, adjacent random states are generated from the previous iteration point. The acceptance criterion determined by the control parameters determines the choice of this new state and thus forms a random Markov chain with a certain length. Second, slowly reduce the control parameters and improve the acceptance criteria until the control parameters tend to zero. The state chain is stable in the optimal state of the optimization problem, thus improving the reliability of the global optimal solution of the simulated annealing algorithm. And also adopt the step search method to propose the group search optimizer algorithm with Metropolis and predictive (MPGSO); secondly, by combining multi-threading and path random stitching, the algorithm is applied to path planning by combining multi-threading and path random splicing techniques, which provides a new method for path planning in complex animation environment.

The model in this paper has three types of population members: the first type, as the discoverer with the best position; the second type, the joiners who approach toward the discoverer with the best position; and the third type, the wanderers who wander randomly in the domain. During each iteration, the individual with the best current position is selected as the discoverer of this iteration, and the MPGSO algorithm is based on the Metropolis criterion and effective improvement on the idea of band trend prediction; the joiners approach the discoverer according to a certain update strategy; the wanderers wander and scan in a certain area. In this paper, a stepwise search approach is used, and joiners and wanderers retain the better solution. The algorithmic ideas are introduced for discoverers, joiners, and wanderers according to the group members' role intelligence.

#### 4.2.1. Discoverer

If the discoverer falls into a certain local extremum, it is easy to cause the whole to fall into a local optimum and the performance of finding the best is degraded. For this reason, the MPGSO algorithm uses the idea of trend prediction to update the experience and position according to the following formula.

(4) $$\begin{array}{c} V_{i}^{k}=c_{1}V_{i}^{k-1}+c_{2}r_{1}\left(X_{\text{bett}}^{k}-X_{\text{best}}^{k-1}\right)\text{,} \end{array}$$

(5) $$\begin{array}{c} X_{i}^{k}=X_{\text{best}}^{k}+r_{2}V_{i}^{k}\text{,} \end{array}$$

where *X*_*i*_^*k*^ ∈ *R*^*u*^ is the position of the ith cluster member in the *k* iteration; *V*_*i*_^*k*^ ∈ *R*^*u*^ is the experience of the ith cluster member in the kth iteration; *X*_best_^*k*^ is the position of the optimal cluster member (discoverer) in the kth iteration; *c*_1_ and *c*_2_ are constant coefficients; *r*_1_ is a randomly distributed [0, 1] vector.

Subsequently, the algorithm determines whether the updated *X*_*i*_^*k*^ is used as the new position of the cluster member in this iteration according to the Metropolis criterion.

The incremental value of the superiority of the Metropolis criterion is calculated according to the following equation

(6) $$\begin{array}{c} \Delta f=f\left(X_{i}^{k}\right)-f\left(X_{i}^{k-1}\right)\text{,} \end{array}$$

where *f*(*X*) is the objective function. If Δ*f* < 0, then accept *X*_*i*_^*k*^ as the new position at the kth iteration of the ith cluster member; otherwise accept *X*_*i*_^*k*^ as the new position at the *k*th iteration of the ith cluster member with the probability derived from (7). In this way, the good moving direction can be saved as experience to predict a better moving position, and the purpose of effectively jumping out of the local minimal value point can also be achieved.

(7) $$\begin{array}{c} \exp\left(-\frac{\Delta f}{MaxIter}\right)\text{.} \end{array}$$

#### 4.2.2. Joiners

The randomly selected very small number of accessions is used with trend prediction idea to update their positions according to (4) and (5), and the remaining accessions updated their positions according to equation (4-2) below.

(8) $$\begin{array}{c} X_{i}^{k}=X_{i}^{k-1}+r_{3}\left(X_{\text{best}}^{k}-X_{i}^{k-1}\right)\text{,} \end{array}$$

where *r*_3_ is a randomly distributed [0, 1] vector. If equation (9) holds, then no update is selected, i.e., *X*_*i*_^*k*^=*X*_*i*_^*k*−1^. Otherwise, update the position.

(9) $$\begin{array}{c} f\left(X_{i}^{k}\right)-f\left(X_{i}^{k-1}\right)>0\text{.} \end{array}$$

#### 4.2.3. Wanderers

The variance calculation was performed in random steps according to equation ().

(10) $$\begin{array}{c} X_{i}^{k}=X_{i}^{k-1}+r_{4}\cdot step\;mutationflag\text{,}\\ mutationflag=r_{5}<\frac{1.5}{n}+{\left(\frac{4n}{k}\right)}^{2}\text{,} \end{array}$$

where *r*_4_, *r*_5_ are *n*-dimensional [0, 1] uniformly distributed vectors, and mutation flag is a Boolean value that marks whether each dimension is mutated or not. For the newly generated *X*_*i*_^*k*^, if equation (11) holds then no update is selected, i.e., *X*_*i*_^*k*^ = *X*_*i*_^*k*−1^. Otherwise, the position is updated.

(11) $$\begin{array}{c} f\left(X_{i}^{k}\right)-f\left(X_{i}^{k-1}\right)>0\text{.} \end{array}$$

## 5. Algorithm Simulation

In order to verify the feasibility and efficiency of the model and algorithm proposed in this paper, the algorithm performance will be tested next using the function optimization problem and compared with genetic algorithm (GA), particle swarm optimization (PSO), group search optimizer (GSO), and group search optimizer algorithm with predictive (PGSO) are compared to prove that the scheme proposed in this paper has better results.

In this paper, three standard test functions are used to test the simulation of the notational algorithm. Among them, the sphere function and Rosenbrock function are used to test the optimization performance of the algorithm for unimodal problems, and the Rastrigin function is used to test the optimization performance of the algorithm for multimodal functions, denoted by *f*_1_(*x*), *f*_2_(*x*), *f*_3_(*x*), respectively.

**Figure 3:**
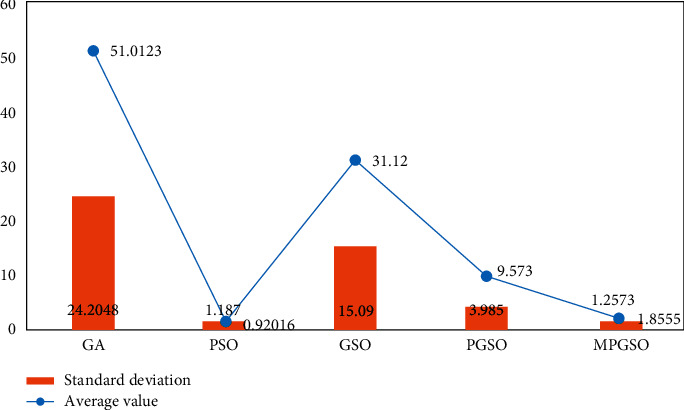
Mean and standard deviation of the results of 50 runs of the sphere function in the low-dimensional case.

**Figure 4:**
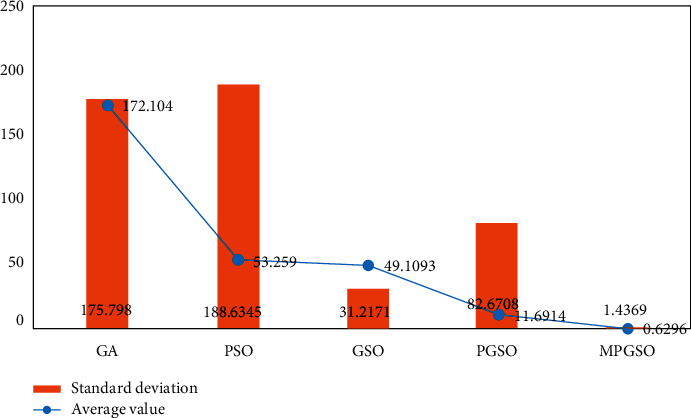
Mean and standard deviation of the results of 50 runs of the Rosenbrock function in the low-dimensional case.

**Figure 5:**
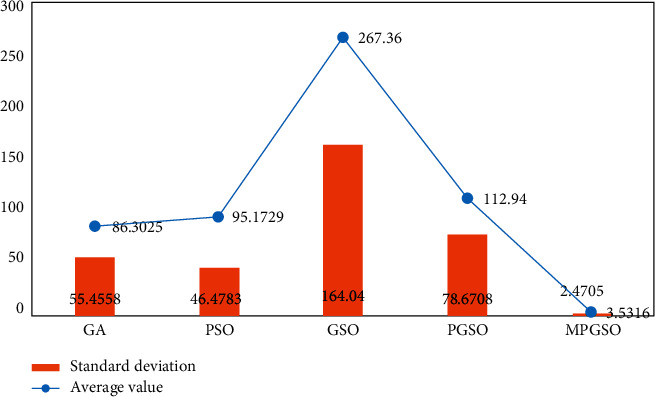
Mean and standard deviation of the results of 50 runs of the Rastrigin function in the low-dimensional case.

The population size is set to 50. When the dimension is 30, the number of iterations is 3000, each result is run 50 times, and the mean and standard deviation of the results are taken for comparison as shown in Figures 3–5.

**Figure 6:**
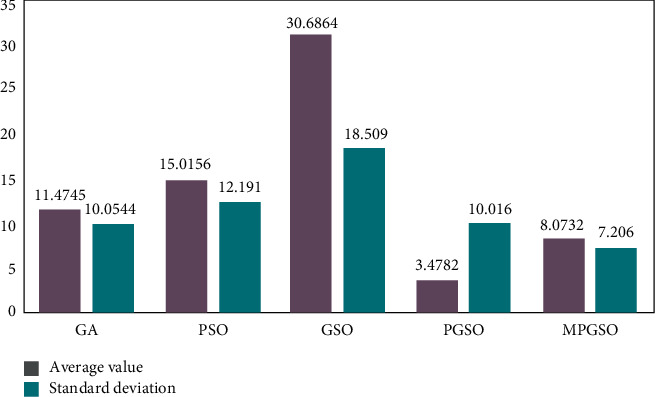
Mean and standard deviation of the results of 10 runs of the sphere function in the high-dimensional case.

**Figure 7:**
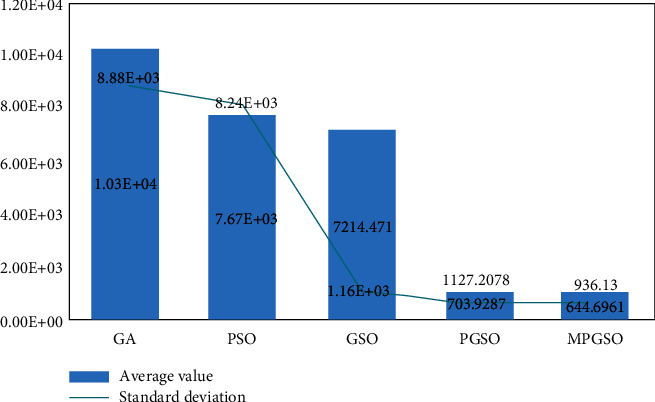
Mean and standard deviation of the results of 10 runs of the Rosenbrock function in the high-dimensional case.

**Figure 8:**
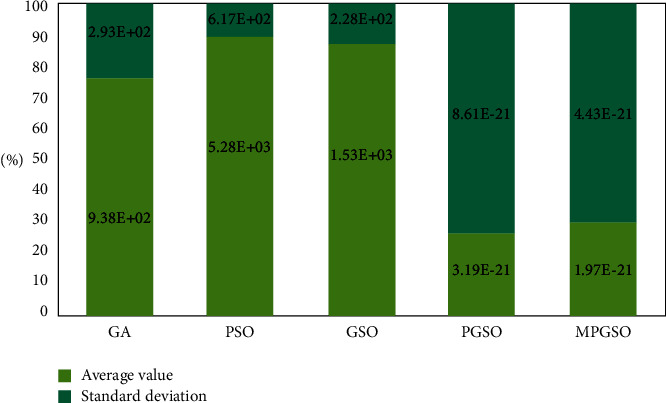
Mean and standard deviation of the results of 10 runs of the Rastrigin function in the high-dimensional case.

The population size is set to 50. When the dimension is 300, the iteration is 60,000 at this point, each result is run 10 times, and the mean and standard deviation of the results are taken for comparison. This is shown in Figures 6–8.

**Figure 9:**
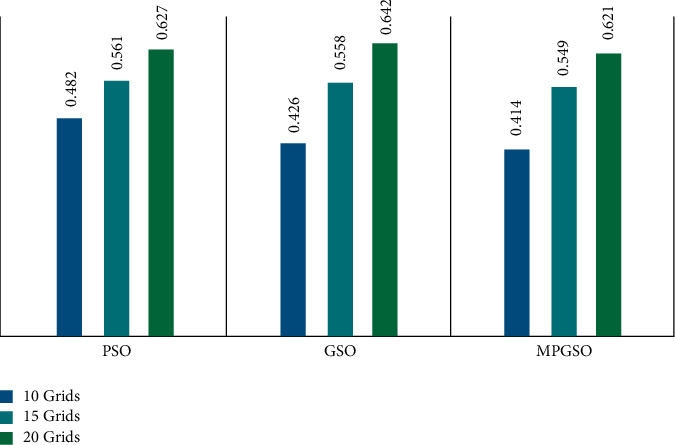
Comparison of path planning time before and after using random path splicing technique.

It can be seen that for the three test functions, MPGSO has better search results than GA, PSO, GSO, and PGSO in the low- and high-dimensional cases, and the stability of the algorithm is higher. In order to more clearly prove the effectiveness of the scheme proposed in this paper, the time of path planning of MPGSO algorithm with standard GSO algorithm and PSO algorithm is calculated separately under the same outer path planning and using random path planning principle, as shown in Figure 9.

The above figure shows that when the general sequential path stitching is used, not only the phenomenon of faster and slower is seen and the population is lost, but also the total path planning time is affected and the path planning efficiency is reduced. After adopting the random path splicing technique, the totality of the particle population and the realism of the motion are effectively improved.

## 6. Conclusion

In the field of group animation creation, the artificial life method of computer animation overcomes the defects of traditional animation creation techniques, such as tedious detailing of movements, difficulty in guaranteeing natural realism, lack of autonomy of animated characters, and heavy workload of animators, and creates a series of excellent works similar to “artificial fish” animation, which greatly improves the efficiency of group animation creation. The efficiency of group animation creation has been greatly improved. However, due to the increasing complexity of the animation character modeling technology used in this method, the degree of woe between the models of the animation system is also increasing, which makes the animation creation increasingly difficult. In particular, when the number of intelligent characters' increases, the computation will increase rapidly in a nonlinear manner, which greatly affects the real-time animation creation and limits the wide-scale application of this method.

In view of this situation, how to reduce the scourge between the models of animation system, reduce the amount of computer operations, and improve the animation creation efficiency without reducing the animation effect and real time has become a hot research topic for the creators of computer group animation. In this paper, we have designed a two-layer decomposed group animation character model based on autonomous intelligences and a “spatial separation” perception algorithm, which effectively improves the perception performance of characters in complex living environments. Based on this, we propose an improved GSO algorithm—MPGSO, apply it to multi-threaded path planning by improving the group search algorithm in terms of search efficiency and ease of implementation, and apply it to the path planning of long paths and complex environments in group animation, where multi-threading and path random splicing techniques are introduced for hierarchical path planning. The implementation of path planning in group animation provides a new idea.

In this paper, we have conducted an in-depth study on the group intelligent character model and achieved certain results, but there is still a lot of work to be done in higher level research and concrete implementation; especially, the following three issues deserve further focus: the performance improvement problem of perception algorithm; the study of behavioral fidelity of animated characters; and the study of higher biological cognitive model.

## Data Availability

The data used to support the findings of this study are included within the article.
